# Back pain and its association with mental health issues in young doctors

**DOI:** 10.1192/j.eurpsy.2024.1350

**Published:** 2024-08-27

**Authors:** M. A. Ghrab, I. Sellami, A. Feki, H. Daoud, A. Abbes, A. Haddar, K. Jmal Hammami, M. Hajjaji, M. L. Masmoudi

**Affiliations:** ^1^Occupational Medicine, Hedi Chaker University Hospital; ^2^Faculty of Medicine; ^3^Rheumatology, Hedi Chaker University Hospital, Sfax, Tunisia

## Abstract

**Introduction:**

Among young medical practitioners, the exigencies of daily practice entail many challenges concerning both physical and mental dimensions. The study of the interplay between these two dimensions is crucial to provide the necessary care for this population.

**Objectives:**

This study aims to evaluate the prevalence of back pain and its association with mental health issues in young medical practitioners.

**Methods:**

A cross-sectional study (January to April 2023) was conducted in the university interns and residents from Sfax. The Nordic questionnaire was used to evaluate back pain. The Generalized-Anxiety-Disorder (GAD -7) and the Patient-Health-Questionnaire (PHQ-9) were used to assess signs of anxiety and depression respectively.

**Results:**

Our population consisted of 404 young doctors. One-hundred and twenty were males with a sex-ratio of 0.42. Among them, 76 (18.8%) had surgical specialties. Mean age was 28.03±2.89, BMI’s mean was 23.65±3.98. Medical history was reported by 29.2% and psychiatric history by 4.9%. The median of the PHQ-9 and GAD-7 score were 3.5 (IQ: [1;6]) and 2 (IQ: [0; 5]). Signs of depression were found in 11.1% of the population whereas anxiety was found in 8.4% of them. Sixty-seven residents (16.6%) reported having back pain in the previous year. Neck pain, upper-back pain and lower back pain were experienced by 8.7%, 6.4% and 10.1% respectively.

Bivariate analysis showed that back pain was associated with PHQ-9 score (p=0.006), GAD-7 score (p=0.018) and it was not associated with BMI (p=0.769) neither with surgical specialties (p=0.824). Lower Back pain was associated with GAD-7 score (p=0.004).

**Conclusions:**

Our study highlights the link between back pain and mental health problems in young doctors. Interventions englobing a better understanding of these two facets are needed to ensure an optimal care for this young population.

**Disclosure of Interest:**

None Declared

